# Post-Translational Modification and Subcellular Distribution of Rac1: An Update

**DOI:** 10.3390/cells7120263

**Published:** 2018-12-11

**Authors:** Abdalla Abdrabou, Zhixiang Wang

**Affiliations:** Department of Medical Genetics, and Signal Transduction Research Group, Faculty of Medicine and Dentistry, University of Alberta, Edmonton, AB T6G 2H7, Canada; abdrabou@ualberta.ca

**Keywords:** Rho GTPases, Rac1, subcellular distribution, nuclear localization, post-translational modification, lipidation, phosphorylation, ubiquitination

## Abstract

Rac1 is a small GTPase that belongs to the Rho family. The Rho family of small GTPases is a subfamily of the Ras superfamily. The Rho family of GTPases mediate a plethora of cellular effects, including regulation of cytoarchitecture, cell size, cell adhesion, cell polarity, cell motility, proliferation, apoptosis/survival, and membrane trafficking. The cycling of Rac1 between the GTP (guanosine triphosphate)- and GDP (guanosine diphosphate)-bound states is essential for effective signal flow to elicit downstream biological functions. The cycle between inactive and active forms is controlled by three classes of regulatory proteins: Guanine nucleotide exchange factors (GEFs), GTPase-activating proteins (GAPs), and guanine-nucleotide-dissociation inhibitors (GDIs). Other modifications include RNA splicing and microRNAs; various post-translational modifications have also been shown to regulate the activity and function of Rac1. The reported post-translational modifications include lipidation, ubiquitination, phosphorylation, and adenylylation, which have all been shown to play important roles in the regulation of Rac1 and other Rho GTPases. Moreover, the Rac1 activity and function are regulated by its subcellular distribution and translocation. This review focused on the most recent progress in Rac1 research, especially in the area of post-translational modification and subcellular distribution and translocation.

## 1. Introduction

The Rho family of GTPases mediates a plethora of cellular effects, including regulation of cytoarchitecture, cell size, cell adhesion, cell polarity, cell motility, proliferation, apoptosis/survival, and membrane trafficking [1]. The Rho family of GTPases accounts for as many as 23 members. Among them, the archetypes RhoA, Rac1, and Cdc42 have been the best characterized [2,3]. Like all members of the small GTPases superfamily, Rho proteins act as molecular switches to control cellular processes by cycling between active, GTP-bound and inactive, GDP-bound states. A major function of Rho proteins is to control cell cytoskeleton remodeling and cell migration. Rac1 regulates actin polymerization and the formation of lamellipodia and membrane ruffles, presumably through interaction with the WASP-family verprolin-homologous (WAVE) complex [4]. Endothelial-specific knockout of Rac1 causes embryonic lethality in midgestation (around E9.5) [5,6].

As a consequence of a large number of key functions assigned to Rho proteins, it is not surprising that they play important roles in many human diseases. Accumulating evidence has implicated Rho GTPases in many aspects of cancer development, especially in cancer cell invasion and metastasis. Deregulated Rho GTPases have been discovered in many human tumors, including colon, breast, lung, myeloma, and head and neck squamous-cell carcinoma [7,8,9,10,11,12,13]. Thus, Rho GTPases and the signal pathways regulated by them have been proposed as potential anticancer therapeutic targets [14]. Like other Rho GTPases, Rac1 is involved in the acquisition of all the hallmarks of cancer [15,16]. Rac1 is overexpressed or mutated in breast cancer and many other cancers [16] and is linked to many other diseases [17]. Due to its well-established role in controlling cytoskeleton dynamics, studies have been focused on revealing the role of Rac1 in cancer metastasis [16,18,19,20]. Until recently, Rac1 itself was considered “undruggable” by small molecules due to the lack of any deep hydrophobic pockets on the surface and earlier failure in finding effective pharmacologic inhibitors [21] The main approach was to target its downstream effectors of kinases such as PAK [22].

The cycling of Rac1 between the GTP- and GDP-bound states is essential for effective signal flow to elicit downstream biological functions [23,24]. Other modifications include RNA splicing [25,26] and microRNAs [27]; various post-translational modifications have also been shown to regulate the activity and function of Rac1 [28]. The reported post-translational modifications include lipidation, ubiquitination, phosphorylation, and adenylylation [29]. Additionally, the Rac1 activity and function are regulated by its subcellular distribution and translocation. This review focused on the most recent progress in Rac1 research, especially in the area of post-translational modification and subcellular distribution and translocation.

## 2. Rho Family of GTPases, Rac Subfamily and Rac1

**Figure 1:**
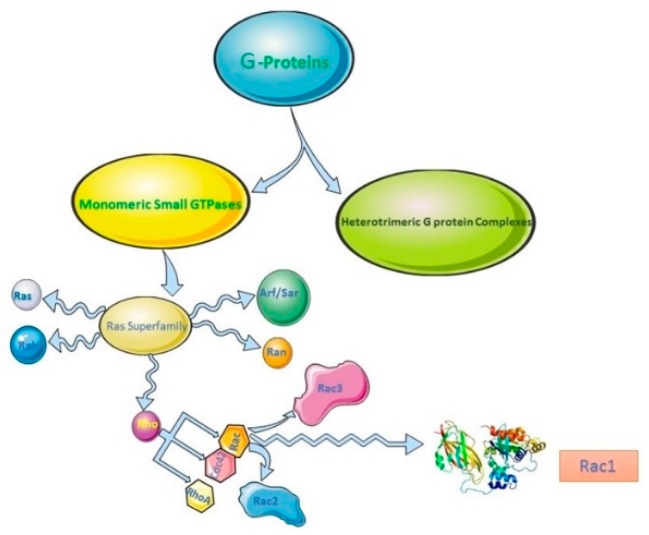
The diagram to illustrate the composition of Ras superfamily.

Rac1 is a small GTPase (M 21,450 kDa) that belongs to the Rho family of GTPases [30,31]. The Rho family of small GTPases is a subfamily of the Ras superfamily. The Ras superfamily is comprised of five subfamilies based on their sequence homologies, which includes the Ras GTPase subfamily, Rho GTPases, the Rab subfamily, Arf subfamilies, and the Ran subfamily [32,33] (Figure 1). Each subfamily functions mostly at distinct subcellular localization and is regulated by its own regulators including Guanine nucleotide exchange factors (GEFs) and GTPase-activating proteins (GAPs).

The Rho family of GTPases is composed of 20 members in mammalian cells [34,35]. The most studied members are the canonical proteins Rho, Rac, and Cdc42 [8,36,37,38,39]. Rho GTPases signal to the cytoskeleton and to vesicular traffic, notably by regulating the dynamics of actin [4,40]. Moreover, Rho GTPases regulate cell size, cell proliferation, cell survival, membrane trafficking, cell motility, polarity, and adhesion [31,32,41]. Like other small GTPases, most Rho family proteins function as switches by cycling between inactive (GDP-bound) and active (GTP-bound) forms. The cycle between inactive and active forms is controlled by three classes of regulatory proteins: GEFs that activate Rho GTPases by promoting the release of GDP to allow binding of GTP; GAPs that inactivate Rho GTPases by stimulating its intrinsic GTP-hydrolysis activity; and guanine-nucleotide-dissociation inhibitors (GDIs), which interact with the GDP-bound inactive Rho to prevent its exchange to the GTP-bound form and prevent its translocation to the membrane for action [32,33].

Rho GEFs are divided in two unrelated families; one family contains a ~200 amino acid (aa) Dbl-homology domain (DH) and the other family contains a ~400 aa Dock homology region (DHR). Dbl-homology family is the largest and best studied Rho GEF family with at least 70 members [34,42]. The Dbl family of Rho GEFs contains both DH and pleckstrin homology (PH) domains. Most Rho GEF activity is mediated by catalytic DH domains [34]. A major mechanism in the regulation of GEFs is the relief of the intramolecular inhibition caused by the PH domain. GEFs also contain several other functional domains, many of which couple to upstream receptors. Thus, it is not surprising that Rho GEFs and Rho are regulated by receptors including epidermal growth factor (EGF) receptor (EGFR) [32,43,44,45]. Recent studies indicate that the cycling between the GTP- and GDP-bound states might be essential for effective signal flow through Rho GTPases to elicit downstream biological functions, and this could involve the concerted action of all classes of the regulatory proteins [23,24].

Like most of the typical small GTPases, the regulatory cycle of Rac1 is exerted by three distinct families of proteins: GEFs, GAP, and GDIs. So far, more than twenty GEFs have been identified for Rac1, some of them specifically for the Rac family, some less selecive [46,47]. Most of the GEFs that act on Rac1 are in the 60-member Dbl family, including members of the Tiam, Vav, PIX, SWAP-70, and P-Rex families. DOCK180, a member of DOCK family of GEFs, has also been shown to regulate Rac1. DOCK proteins contain a conserved catalytic domain, a phospholipid domain, and a DOCK homology region 2 [46,47]. It is generally accepted that the membrane localization of Rac is a prerequisite for Rac activation [48]. Several Rho GAPs were already known in 1992; however, since then, the number of known genes encoding a Rho-GAP domain has increased to about 80 [24,39]. The functions of most of these proteins are still unknown, though the variations in additional domains found in these proteins indicate that they act in a wide variety of signaling pathways in different tissues. The GDIs are pivotal regulators of Rho GTPase function. GDIs control the access of Rho GTPases to regulatory GEF and GAP, to effectors and to membranes where such effectors reside [49,50].

Rac is also known as Ras-related C3 botulinum toxin substrate. There are three members in the Rac subfamily, including Rac1, Rac2, and Rac3, which share a significant sequence identity (~88%). These three diverge primarily in the C-terminal 15 residues. All the Rac-related proteins stimulate the formation of lamellipodia and membrane ruffles. Rac1 promotes lamellipodia formation by stimulating the polymerization of branched actin at the leading edge of the cell. Activated Rac1 binds to the WASP-family verprolin-homologous (WAVE) proteins through association with its downstream effector insulin receptor tyrosine kinase substrate p53 (IRSp53). The WAVE proteins then bind to and activate the protein complex composed of actin nucleating protein and actin-related protein 2/3 (Arp2/3), which leads to the enhanced polymerization of branched actin at the leading edge and promote lamellipodia formation [19,47,51]. Additionally, Rac1 has also been shown to regulate the expression of various matrix metalloproteinases (MMPs), which are required for the proteolytic degradation of the extracellular matrix [5]. The in vivo and in vitro studies in the last decades have firmly established the role of Rac1 in cancer cell invasion and metastasis [18]. Rac1 can stimulate MMP-1 or MT1-MMP production in lung cancer cell lines and enhance invasion in vitro [52]. When adherens junctions are weakened by EGF or hepatocyte growth factor (HGF), Rac is required to promote cell migration and invasion [53,54].

## 3. Regulation of Rac1 by EGFR and other Membrane Receptors

The activity and the function of Rac1 is ultimately regulated by environmental cues, among which are the membrane receptors including many receptor tyrosine kinases (RTKs) [55]. The most studied membrane receptor in this regard is EGFR. It is well-established that EGFR activates multiple signaling pathways and regulates many cellular functions including cell proliferation, differentiation, survival, adhesion, and migration [56,57,58]. Many EGFR-mediated cell functions are partially mediated by Rac1 and other Rho family proteins. 

The most common mechanism by which EGFR regulates Rac1 activity is through the regulation of Rac1 GEFs [55]. GEFs implicated in EGF-stimulated Rac1 activation include members of Tiam, Sos, DOCK, Vavs, and Asef family [59,60,61,62,63]. EGF stimulates Tiam1 through the activation of Phosphoinositide 3-kinases (PI3K) [64]. Moreover, EGF stimulates two waves of Rac1 activation; first at 5 min and the second at 12 h following the addition of EGF. Cell migration is driven by the second wave of EGF-induced Rac1 activation. It was further shown that EGF activates Rac1 through the activation of Tiam1, either via 14-3-3ζ or through 5-lipoxygenase-leukotriene C4 [65,66]. 

Recent data further supported the role of EGFR in the regulation of Rac1 activity and cell migration by controlling Rac1 GEF. EGFR exerts its effects mostly by phosphorylating Rac1 GEF, but also by ubiquitination and controlling the level and availability. For example, treatment of cells with either EGF or platelet-derived growth factor-stimulated tyrosine phosphorylation and activation of Vav2, which promotes cell migration through the activation of Rac1, Cdc42, and RhoA [67]. EGF stimulates the phosphorylation of Try94 of Asef, which activates Asef [62]. It was further shown that EGF, basic fibroblast growth factor and hepatocyte growth factor all activate Asef through PI3K, which promotes cell migration [63]. It was later reported that EGFRvIII, a constitutively active EGFR mutant, stimulates the phosphorylation of Dock180 via Src, which stimulates Rac1 signaling, glioblastoma cell survival, and migration [68]. Moreover, EGFRvIII induces the phosphorylation of Dock180 via protein kinase A, which activates Rac1 and promotes glioma tumor growth and invasion [69]. 

EGFR may also regulate Rac1 GEF by controlling its level/availability. It was shown that EGFR activates Rac1 by increasing the accumulation of Tiam1 in colon cancer and non-small-cell lung cancer cells. Moreover, the effects of EGFR on the accumulation of Tiam1 are through the activation of Akt. Activated Akt promotes the interaction between the Tiam1 and 14-3-3 protein by phosphorylating Tiam1 [70]. Recently, it was reported that Vav3.1, a shorter member of the Vav family, was down-regulated by the addition of EGF and Transforming growth factor β(TGFβ) in the pathogenesis of oral squamous cell carcinoma [71]. Down-regulation of Asef by siRNA inhibits the EGF-induced activation of Rac1. EGF stimulates the phosphorylation of Try94 of Asef, which activates Asef [62]. The activated EGFR disrupts adherens junctions in human mammary epithelial cells by Vav2 and Rac1/Cdc42 activation [72]. It was further shown that EGF, basic fibroblast growth factor, and hepatocyte growth factor all activate Asef through PI3K, which promotes cell migration [63]. EGF enhances the ubiquitination of Dock180 by stimulating the translocation of both Dock180 and ubiquitin to the plasma membrane (PM), whereas Dock180 is ubiquitinated [73]. It was also reported that the Mer-family tyrosine kinase activation stimulates a post-receptor signaling cascade involving Src-mediated FAK phosphorylation, which promotes the formation of a p130CAS/CrkII/Dock180 complex to activate Rac1 [74]. Most recently, it was shown that EGFR overexpression or expression of EGFRvIII stimulates Rac1 activation and promote glioblastoma cell migration through activating the MLK3-JNK signaling axis [75]. 

Much less is known about the role of EGFR and other membrane receptors in the regulation of Rac1 GAPs [55]. It is known that some Rac-GAPs are stringently regulated by receptor stimulation. For example, the chimaerin Rac-GAPs are activated by diacylglycerol (DAG) generated in response to growth factor receptor activation. DAG binds to chimaerins to promote their redistribution to the PM, where they inactivate Rac1 [76,77]. This regulation is via PKC-mediated phosphorylation and other protein-protein interactions, which modulates the subcellular localization and membrane association of chimaerin [78,79]. It was shown recently that EGFR and Src signaling regulates FilGAP through association with RBM10 [80].

Recently, more studies have demonstrated the role of EGFR-stimulated activation of Rac1 in cancer cell migration, invasion, and survival. EGF stimulates head and neck squamous cell carcinoma by activating PKD1-fibronectin-Rac1/Cdc42 and metalloproteinases (NMP) [81]. It was shown previously that Rac1 can stimulate MMP-1 production in lung cancer cell lines and enhance invasion in vitro [52]. EGF also stimulates PI3K-Akt-Rac1 signaling cascades through the activation of Giα2, which promotes the migration and invasion of prostate cancer cells [82]. Periodic mechanical stress stimulates nucleus pulpous cell proliferation by activating EGFR-Rac1-ERK cascades [83]. Ligand-specific EGFR signaling regulates apicobasal polarity via PI3K and Rac1, which controls cell fate and pancreatic organogenesis [84]. T-cadherin translocation to cell-cell contact is sensitive to EGFR-mediated activation of Rac1 and p38MAPK [85]. In human epidermal growth factor receptor 2 (HER2)-positive breast cancer cells, two distinct mTORC2-dependent pathways converge on Rac1, either through Akt-Tiam1 or through PKC-RhoGDI2 [86]. 

## 4. Regulation of Rac1 Activity by Post-Translational Modification

The cycling of Rac1 between the GTP- and GDP-bound states is essential for effective signal flow to elicit downstream biological functions [23,24]. Other modifications, including RNA splicing [25,26], microRNAs [27], and various post-translational modifications, have also been shown to regulate the activity and function of Rac1. The reported post-translational modifications include lipidation, ubiquitination, phosphorylation, and adenylylation [29], which were all shown to play important roles in the regulation of Rac1 and other Rho GTPases [28]. 

### 4.1. Regulation of Rac1 Activity by Lipidation

**Figure 2:**
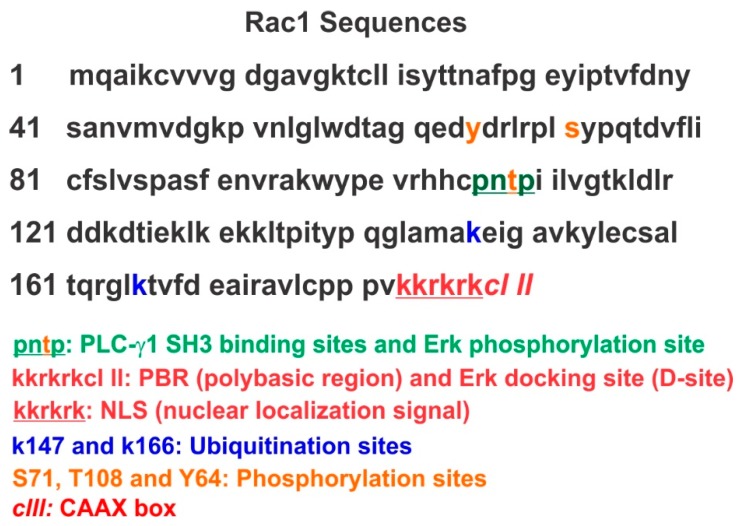
Rac1 sequences, various motifs, and amino acid residues subjected to the post-translational modification.

Protein prenylation involves the attachment of either a 15-carbon farnesyl or 20-carbon geranylgeranyl isoprenoid to a cysteine residue four amino acids from the C-terminus of a protein. Prenylation is the first and most reported post-translational modification of Rac1 and other Rho GTPases, which plays a critical role in the regulation of Rac1 by targeting Rac1 to the PM and facilitating Rac1 interaction with GEFs [87]. Like many other small G proteins, Rac1 has a unique C-terminal amino acid sequence of a CAAL, where C is cysteine, A is an aliphatic amino acid, and L is leucine (Figure 2). Small G proteins having this C-terminal structure were post-translationally processed: (1) geranylgeranylation of the cysteine residue; (2) removal of the A-A-Leu portion; and (3) carboxyl methylation of the exposed cysteine residue. Rac1 has been shown to be geranylgeranylated at its C-terminal Cysteine residue [88]. 

It was initially believed that the newly synthesized Rho GTPases such as Rac1 is quickly prenylated in the cytosol and then moved to Endoplasmic reticulum for further modifications [89]. However, later research indicates that the entrance and passage of small GTPases including Rac1 through the prenylation pathway is regulated by two splice variants of SmgGDS. SmgGDS-558 selectively associates with prenylated small GTPases and facilitates their trafficking to the PM, whereas SmgGDS-607 associates with nonprenylated GTPases and regulates the entry of Rho small GTPases into the prenylation pathway. These results indicated that SmgGDS splice variants can regulate the entrance and passage of small GTPases through the prenylation pathway [89]. 

Interestingly, we recently showed that Rac1 contains an ERK docking site located at its C-terminal 183KKRKRKCLLL192 (Figure 2). The core consensus motif of ERK D-sites is (K/R)1-3-X1-6-φ-X-φ [90,91]. The D-site is critical for the interaction between Rac1 and ERK and for the phosphorylation of Rac1 T108 by ERK [45]. The Rac1 D-site was no longer present following its geranylgeranylation. Thus, the interaction between Rac1 and ERK should occur before the geranylgeranylation. The regulated entry into the prenylation pathway by SmgGDS provides a mechanism to allow the interaction between ERK and non-prenylated Rac1. 

It was late reported that Rac1 is also palmitoylated [92]. Palmitoylation is the post-translational covalent binding of the 16-carbon fatty acid palmitate through a thioester bond. Different from prenylation, palmitoylation is reversible [93]. There is no conserved motif identified for palmitoylation. In Rac1, among the two possible Cys for palmitoylation, Cys6 (N-terminus), or Cys178 (C-terminus), Cys178 was shown to be palmitoylated. Blocking Rac1 palmitoylation by mutation significantly reduces PM localization and the GTP loading of Rac1, which leads to a reduced activation of PAK at the PM [92].

### 4.2. Rac1 Ubiquitination

The expression level of Rho GTPase is also an important factor in regulating its signaling and function. Like most proteins, the expression level of Rho GTPases is determined by both its synthesis and degradation. The synthesis of Rac1 and the other Rho GTPases are mostly regulated by various external stimuli. The degradation of Rac1 and the other Rho GTPases are regulated through their ubiquitination and subsequent proteasomal degradation [28]. In Rho GTPases, the positions of ubiquitinated lysines are not conserved. While the most identified ubiquitinated lysines are located in a region on the opposite side of the GTP-binding and switch regions, many additional ubiquitinated lysines are scattered around the protein surface. It has been well documented that Rac1 is ubiquitylated by E3 proteins HACE, XIAP, and c-IAP1 on K147, and by FBXL19 on K166 dependent on S71 phosphorylation [94,95,96]. Rac3 is ubiquitylated on K166 by the E3 protein FBXL19 [97].

The significance of Rac1 ubiquitylation was revealed by the discovery of HACE1 ubiquitin ligase as a tumor suppressor. While HACE1 has multiple targets, it has been shown that loss of HACE1 increases Rac1 activity, which induces reactive oxygen species generation and cell migration that likely contribute to Rac-mediated tumor progression [28,98].

### 4.3. Phosphorylation of Rac1 and other Rho GTPases

Recent findings have suggested that additional regulatory mechanisms such as phosphorylation might further contribute to the tight regulation of Rho GTPases [99]. Rac1 is phosphorylated on S71 by Akt [100] (Figure 2). This phosphorylation of Rac1 inhibits its GTP binding activity without any significant change in GTPase activity. Both the GTP-binding and GTPase activities of the mutant Rac1 S71A are abolished regardless of the activity of Akt [100]. It was later reported that the phosphorylation of Rac1 S71 decreases the pathogenic effect mediated by Clostridium difficile toxin A (TcdA) [101]. Moreover, phosphorylation of Rac1 at S71 represents a reversible mechanism to determine the binding specificity of Rac1/Cdc32 to their downstream substrates [102]. In addition, Rac1 is phosphorylated at Y64 by FAK and Src kinases (Figure 2); Y64 phosphorylation targets Rac1 to focal adhesions. Rac1-Y64F displayed increased GTP-binding, increased association with βPIX, and reduced binding with RhoGDI as compared with wild-type Rac1. Rac1-Y64D had less binding to PAK than Rac1-WT or Rac1-64F. In vitro assays demonstrated that Y64 in Rac1 is a target for FAK and Src [103]. These findings demonstrate that both Serine/Threonine and tyrosine phosphorylation of Rac1 are common phenomena and regulate multiple aspects of Rac1 functions. So far, there is no pathogenic evidence to support the role of these phosphorylations in the development of cancer and other diseases. Further research is needed.

Recently, we identified that PLC-γ1 is a Rac1 GEF both in vitro and in vivo [43]. We showed that the interaction between PLC-γ1 and Rac1 is mediated by PLC-γ1 SH3 domain and Rac1 proline-rich motif 106PNTP109 [43]. Moreover, we showed that EGF-induced interaction between the PLC-γ1 SH3 domain and the Rac1 106PNTP109 motif resulted in the activation of Rac1 and enhanced EGF-induced cytoskeleton reorganization and cell migration. Interestingly, sequence analysis of Rac1 shows that Rac1 T108 within the 106PNTP109 motif is likely an ERK phosphorylation site; Rac1 has the ERK docking site 183KKRKRKCLLL192 (D-site) at C-terminus (Figure 2). We showed that both transfected and endogenous Rac1 interacts with ERK and this interaction is mediated by its D-site [45]. GFP-Rac1 is threonine (T) phosphorylated in response to EGF, and EGF-induced Rac1 threonine phosphorylation is dependent on the activation of ERK. Furthermore, mutant Rac1 with the mutation of T108 to alanine (A) is not threonine phosphorylated in response to EGF. In vitro ERK kinase assay further shows that pure active ERK phosphorylates purified Rac1, but not mutant Rac1T108A. Additionally, we showed that Rac1 T108 phosphorylation decreases its activity, partially due to inhibiting its interaction with PLC-γ1. T108 phosphorylation targets Rac1 to the nucleus, which isolates Rac1 from other GEFs and hinders Rac1’s role in cell migration. We concluded that Rac1 T108 is phosphorylated by ERK in response to EGF, which plays an important role in regulating Rac1 [45].

As many of the kinases, such as Akt, ERK, and Src, that phosphorylates Rac1 are activated by EGFR and other RTKs, it is not surprising that EGFR and other RTKs play important roles in the regulation of Rac1 phosphorylation. We have shown that EGF stimulates the Rac1 T108 phosphorylation [44].

## 5. Regulation of Subcellular Localization of Rac1

It has been well documented that Rac1 and other Rho GTPases distribute between multiple cellular compartments. At various subcellular locations, Rho GTPases may interact with different effectors, and thus, produce spatially complex signaling outputs [104]. Besides the well-documented localization in the cytosol and the PM, Rac1 has been shown to localize to the other subcellular compartments, including endosomes [105] and the nucleus [106]. The most studied translocation of Rac1 has been its translocation to the PM and to the nucleus.

### 5.1. Regulation of the PM Localization of Rac1

In addition to cycling between GDP bound inactive form to GTP-bound active form, Rho GTPases also cycle between PM and cytosol, which regulates their activities and function in actin cytoskeleton remodeling and cell migration [104]. The lipidation of Rac1 is important for its membrane-binding. It was shown that membrane-bound Rac1 is geranylgeranylated and the soluble Rac1 is not geranylgeranylated [107]. In the aged mouse brain, the reduced geranylgeranylation of Rac1 due to lower geranylgeranyltransferase activity decreases the PM association of Rac1 [108]. 

The subcellular localization of Rac1 is also regulated by its C-terminal polybasic region (PBR) (Figure 2). Many small GTPases in the Ras and Rho families have a PBR comprised of multiple lysines or arginines. The PBR controls diverse functions of these small GTPases, including their ability to associate with membranes, interact with specific proteins, and localize in subcellular compartments. A major function of the PBR is to promote the interactions of small GTPases with the guanine nucleotide exchange factor SmgGDS [109].

Palmitoylation of Rac1 targets Rac1 to the lipid raft, an ordered membrane region, of the PM [92]. This is a very important finding, as Rac1 and RhoA have been shown to be localized in caveolar, and the cholesterol-enriched lipid rafts are the major site of signaling by Rac1 and RhoA [110,111,112]. On the other hand, palmitoyl modification is known to target proteins to lipid rafts. For example, H-Ras is targeted to lipid rafts due to its palmitoylation [113]. Thus, the reported palmitoylation of Rac1 provides a mechanism for the localization of Rac1 in lipid rafts [114]. The cycle between palmitoylation and depalmitoylation allow proteins to transiently associate with membranes, thereby regulating their sorting, localization, and function [93]. Rac1 palmitoylation requires its prior prenylation and the presence of the intact PBR. Palmitoylation is also essential for Rac1-mediated cell spreading and migration [92]. It was further shown that inhibition of Rac1 palmitoylation significantly reduces the PM association of Rac1 [115].

### 5.2. Regulation of the Nuclear Localization of Rac1

Rac1 also localizes to endosomes [105] and the nucleus [106]. Rac1 activation on early endosomes and subsequent recycling of Rac1 to the PM ensure polarized signaling, leading to localized actin-based migratory protrusions [105] and spatial restriction of Rac1 motogenic signals, which promotes mesenchymal motility. In the nucleus, the accumulation of Rac1 has been linked to regulation of Rac1 proteasomal degradation [116]. Additionally, cell-cycle-dependent accumulation of nuclear Rac1 promotes mitotic progression [117]. These findings suggest that nucleocytoplasmic shuttling is important for the spatial control of specific Rac1 functions. 

Both Rac1 and RhoA have been found to be localized to the nucleus [44,109,117,118]. The data regarding the mechanisms regulating Rac1 nuclear localization are very controversial. It was shown that the PBR of Rac1 has a functional nuclear localization signal (NLS) [119] (Figure 2). However, PBR has also been shown to target Rac1 to the PM. It was found that the Rac1 NLS is cryptic in the sense that it is inhibited by the adjacent geranylgeranyl modification. However, the same research showed that the endogenous nuclear Rac1 is pranylated [117]. It was also reported that the nuclear import of Rac1 is mediated by the direct interaction with karyopherin α2 and Rac1 activation is required for its nuclear localization [120]. In a study of Rac1 palmitoylation, it is found that C6 is not palmitoylated, but mutation of C6 to S reduced nuclear localization of Rac1 [92]. It is not clear how C6 mutation alters the nuclear localization of Rac1.

We have shown that Rac1 T108 phosphorylation by ERK targets Rac1 to the nucleus [44]. We show by both fluorescence microscopy and Western blotting that significant amount of GFP-Rac1 is translocated from cytosol to the nucleus in response to EGF. Inhibition of T108 phosphorylation by mutating T to A blocks the nuclear localization of Rac1. Moreover, the T108 phosphorylation mimic GFP-Rac1T108E was almost exclusively localized to the nucleus with or without EGF stimulation [44]. 

Although the majority of RhoA is localized in the cytosol and at the PM of cells, a fraction of the total RhoA pool has been shown to be distributed to the nucleus and regulates downstream signaling [121,122,123,124,125]. We showed that RhoA is phosphorylated by ERK at S88. While this phosphorylation increases the PM translocation of RhoA, it does not target RhoA to the nucleus [45]. 

Like other members in the Rho proteins family, both Rac1 and RhoA have a PBR in their C-termini. The PBR is adjacent to and immediately precedes the C-terminal CAAX sequence. In addition to the prenylation of the CAAX motif and the interaction with RhoGDI, the subcellular localization of Rac1and RhoA is also regulated by its PBR. It has been shown that the Rac1 PBR (PVKKRKRK) contains an NLS, and thus, promotes Rac1 nuclear accumulation, whereas the RhoA PBR (RRGKKKSG) lacks an NLS and sequesters RhoA in the cytosol [116]. 

Recently, we examined the relative contribution of ERK-induced phosphorylation and the PBR on the subcellular localization of Rac1 and RhoA. We showed that the PBR is the determining factor for the subcellular localization of both Rac1 and RhoA. Replacing RhoA PBR with Rac1 PBR resulted in significant nuclear localization of RhoA regardless of the phosphorylation status of RhoA. On the other hand, RhoA PBR was able to significantly reduce the nuclear localization of Rac1 regardless of the phosphorylation status of Rac1 [45]. EGF-induced nuclear translocation of Rac1 is dependent on ERK-induced Rac1 T108 phosphorylation [44]. However, EGF-induced nuclear translocation of Rac1 also requires the presence of Rac1 PBR [45]. It has been shown that the switching of the PBRs between Rac1 and RhoA alters their nuclear accumulation [116]. 

While it is well documented that a portion of Rac1 is localized to the nucleus and some mechanisms underlying the nuclear localization of Rac1 have been revealed, very little is known regarding the function and biological significance of nuclear Rac1. Nuclear localization of Rac1 could serve as a very important mechanism regulating the activity and function of Rac1. Localization into the nucleus will isolate Rac1 from its regulatory proteins localized in the cytoplasm and the PM. Currently, there is no report of the nuclear localization of Rac1 GEFs. Most of the known Rac1 GEFs, including PLC-γ1, Vav proteins, and Tiam1, are outside of the nucleus [126,127,128,129], and thus, will not be able to activate the nuclear Rac1. On the other hand, localization of Rac1 to the nucleus allows its interaction with a very different set of molecules and carry out different functions. It was shown that Rac1 regulates the activity of nuclear factor-κB (NF-κB) [130]. Rac1 also regulates gene transcription by interacting with STAT3 and STAT5 [131,132]. In the nucleus, the accumulation of Rac1 has been shown to regulate the proteasomal degradation of Rac1 [116]. It was also reported that Rac1 is localized to the nucleus during the G2 phase of the cell cycle and promotes cell division [117]. Recently, nucleolar phosphoprotein B23 was identified as a nuclear binding partner of Rac1; this binding depends on the N-terminal 88 amino acids of Rac1 [133]. Rac1 nuclear exit depends on two nuclear export signals (NES) and interaction with B23. Activated Rac1 in the nucleus modulates nuclear actin polymerization and nuclear membrane shape, which controls cell invasion [133]. However, our understanding of the function and biological significance of nuclear Rac1 is still very limited, and further research is needed.

## 6. Conclusions

**Figure 3:**
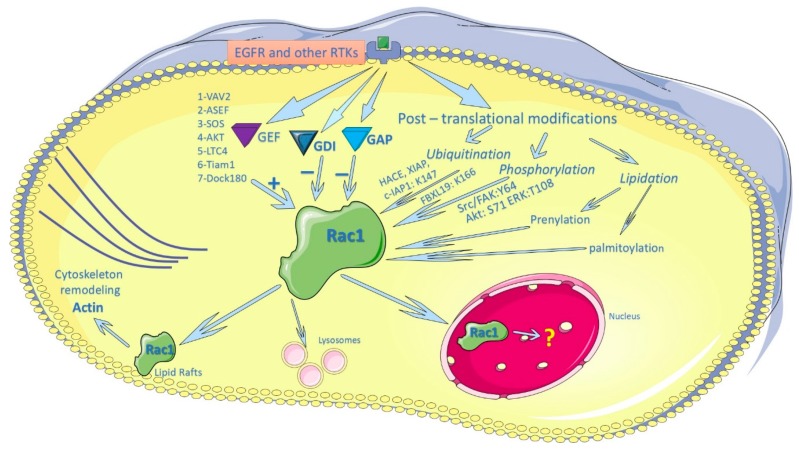
Summary of Rac1 regulation, subcellular distribution, and function.

As shown in Figure 3, while Rac1 activity is primarily regulated by GEF, GAPs, and GDIs, post-translational modification provides additional mechanisms for the regulation of Rac1 activity and function. Recent research on Rac1 and other Rho GTPases has been concentrated on their post-translational modification and subcellular distribution. Recent progress includes the discovery of multiple novel post-translational modifications of Rac1 such as the palmitoylation, phosphorylation, and ubiquitination of Rac1. Besides shuttling between PM and cytoplasm, Rac1 also shuttles between nucleus and cytoplasm in response to EGF and other stimuli. The nuclear localization of Rac1 is regulated by both the NES and NLS motifs and the various post-translational modifications. Very little is known regarding the function and biological significance of Rac1, which needs further research (Figure 3).
